# Characterization of Vascular Patterns Associated with Endothelial Glycocalyx Damage in Early- and Late-Onset Preeclampsia

**DOI:** 10.3390/biomedicines10112790

**Published:** 2022-11-02

**Authors:** Marina M. Ziganshina, Kamilla T. Muminova, Nailia R. Khasbiullina, Zulfiya S. Khodzhaeva, Ekaterina L. Yarotskaya, Gennady T. Sukhikh

**Affiliations:** 1 National Medical Research Center for Obstetrics, Gynecology and Perinatology of the Ministry of Health of the Russian Federation, Oparina Street 4, Moscow 117997, Russia; 2 Department of Obstetrics, Gynecology, Perinatology and Reproductology, Institute of Professional Education, I.M. Sechenov First Moscow State Medical University, Trubetskaya Street 8-2, Moscow 119991, Russia

**Keywords:** pregnancy, early-onset preeclampsia, late-onset preeclampsia, vascular patterns, BPLab 24 h blood pressure monitoring, endothelial glycocalyx, hyaluronic acid (HA), heparan sulfate proteoglycans (HSPGs), syndecan 1 (SDC 1)

## Abstract

This paper provides an assessment of molecular and functional changes in blood vessels, and a description of vascular patterns during preeclampsia (PE). Patients with normal pregnancy, and pregnancy complicated by PE at earlier (20–34 weeks) and later terms (≥34 weeks) underwent a 24 h monitoring of blood pressure, central hemodynamics, arterial stiffness, and myocardial function. The blood levels of the structural components of endothelial glycocalyx (eGC): syndecan-1 (SDC 1), heparan sulfate proteoglycan 2 (HSPG2), and hyaluronic acid (HA) were determined. In early-onset PE, the vascular pattern comprised changes in all structural components of eGCs, including transmembrane proteoglycans levels, and severe disorders of central hemodynamics, arterial stiffness, and myocardial changes, probably leading to more severe course of PE and the formation of morphological grounds for cardiovascular disorders. The vascular pattern in late-onset PE, including changes in HA levels, central hemodynamics, and myocardial function, may be a signal of potential cardiovascular disorder. PE may change adaptive hemodynamic responses to a pathological reaction affecting both arterial elasticity and the left ventricular myocardium, with its subsequent hypertrophy and decompensation, leading to a delayed development of cardiovascular disorders after PE. Further clinical studies of these indicators will possibly identify predictors of PE and long-term consequences of the disease.

## 1. Introduction

Preeclampsia (PE) is a pregnancy-specific condition which may arise after 20 weeks of gestation and manifest with hypertension (≥140/90 mmHg) on two occasions 4 h or more apart, proteinuria, and one or more other disorders: thrombocytopenia, elevated serum creatinine or liver transaminases, kidney or hepatic failure, neurological compromise, fetal growth restriction. Two clinical phenotypes of the disease are distinguished: early-onset PE (20–34 weeks of gestation) and late-onset PE (≥34 weeks). It is believed that early-onset PE develops in the background of the impaired trophoblast invasion and endothelial dysfunction, leading to fetal growth restriction, while late-onset PE is mainly induced by maternal cardio-vascular disorders. Respectively, early-onset PE is associated with a more severe course and increased risk of adverse and sometimes fatal pregnancy outcomes, i.e., placental abruption, stroke, eclampsia, iatrogenic preterm delivery, etc., compared to late-onset PE; at the same time, both forms of the disease lay the grounds for long-term maternal morbidity [1,2].

Despite intensive efforts in studying etiology, pathogenesis, and attempts to improve prediction and timely diagnosis of preeclampsia (PE), the prevalence of the disease has not decreased yet. In fact, according to a number of authors, it tends to increase, reaching 2–8% of the general population [1]. Taking into account PE’s complex pathogenesis, it is generally accepted that, along with placenta and immunology, the key player in development of PE and its complications is the vascular factor, in particular endothelial dysfunction and specific cardiovascular function during pregnancy [2].

The main diagnostic criterion of PE is an increase in blood pressure (BP), but this criterion is not sufficient to clarify and stratify deterioration risks. Noninvasive daily blood pressure monitoring (DBPM) is preferable to routine BP measurements, because this test provides a comprehensive assessment of cardiovascular function based on the ambulatory arterial stiffness and pulse wave velocity and augmentation indexes. These parameters reflect artery rigidity and allow stroke and cardiovascular death prediction [3]. Moreover, during 24 h blood pressure monitoring using the BPLab device, additional parameters can be evaluated, i.e., pulse wave rise velocity, which indirectly correlates with myocardial contractility, total vascular resistance and dynamic load [4,5]. However, all direct and indirect parameters mentioned above reflect functional sequelae of a pathological process resulting from endothelial dysfunction, where the role of endothelial glycocalix (eGC) dysfunction cannot be ignored [6,7].

Endothelial activation with subsequent dysfunction is the result of an enhanced proinflammatory milieu due to placenta ischemia observed in both PE phenotypes (early- and late-onset PE). Proinflammatory stimuli promote damage and destruction of the superficial protective layer localized at the lumen surface of the endothelial cells, i.e., eGC [8,9]. This, in turn, leads to eGC dysfunction, which adversely affects main functions of endothelial cells, namely vascular tone regulation, permeability, adhesive contacts with vascular walls [10]. An increase in the levels of structural components of eGC (hyaluronic acid (HA), heparan sulfate proteoglycans (HSPGs), endocan-1) was demonstrated in cardiovascular diseases [11,12,13], and metabolic disorders [14], proving endothelial dysfunction. Several clinical studies reported a significant increase in serum proteoglycan levels in early- and late-onset PE [15,16]. However, eGC dysfunction is not well-studied in PE; in particular there is no data on a possible correlation between a woman’s hemodynamic profile and eGC dysfunction. Taking into account the major contribution of eGC to vascular tone regulation and elastic characteristics of the endothelial wall, such correlation seems highly probable, since under permanent inflammatory conditions, vascular rigidity increases due to eGC components thinning and shedding [17].

Our study aimed to determine the hemodynamic changes and changes in serum levels of soluble forms of glycocalix proteoglycans in early-onset and late-onset PE, and to reveal the correlation between these parameters. 

## 2. Materials and Methods

### 2.1. Study Population

Patients were matched by age and terms of gestation, because these two parameters are the most common and relevant to the study factors that affect hemodynamics. All patients were divided into 4 groups: (i) early-onset PE (PE1; n = 20); (ii) healthy pregnancy between 20 and 34 weeks (NP1; n = 19); (iii) late-onset PE (PE2; n = 21); and (iv) healthy pregnancy of 34 weeks or onwards (NP2; n = 20). The study was approved by the Ethics Committee of the National Medical Research Center for Obstetrics, Gynecology, and Perinatology named after academician V.I. Kulakov, Moscow, Russia (PR No. 10-04/12/2018). 

### 2.2. Inclusion and Exclusion Criteria

Inclusion criteria for all patients were an age from 18 to 40 years and a spontaneous singleton pregnancy. Clinically diagnosed preeclampsia was an inclusion criterion for PE group patients. For the control groups, the inclusion criteria were: no complications at any term of pregnancy; no chronic disease; no medical therapy (except for vitamins or mineral supplements); normal vaginal flora; normal ultrasonography and Doppler ultrasonography during current pregnancy; term delivery of newborns with normal body weight, length and Apgar score 9–10; without any congenital abnormalities or signs of prenatal fetal distress.

Exclusion criteria for all groups were: multiple pregnancy; chromosomal abnormalities in parents or newborns; severe somatic diseases; autoimmune diseases, including antiphospholipid syndrome; acute and chronic inflammatory diseases; oncological diseases; red blood cell sensitization; history of blood transfusion or organ transplantation; immunoglobulin therapy; and use of drugs which affect antibody production and bioavailability, including low-molecular-weight heparins.

### 2.3. Diagnostic Evaluation of Preeclampsia and Fetal Growth Restriction

Preeclampsia was diagnosed according to international guidelines, as specified in the introduction [1,18].

FGR was diagnosed according to the Delphi consensus of 2016 [19]. Early-onset FGR was determined before the 32nd week of gestation with the estimated fetal weight and/or abdominal circumference (AC) less than the third percentile, or the umbilical artery Doppler with absent and/or zero diastolic flow, or when two of the following three parameters were present: (1) estimated fetal weight and/or AC < the tenth percentile, (2) pulsatility index (PI) of the uterine artery > the 95th percentile, and (3) PI of the umbilical artery > the 95th percentile. Late-onset FGR was determined if estimated fetal weight and/or AC was less than the third percentile or if two of the following indicators were present: (1) estimated fetal weight and/or AC < the tenth percentile, (2) fetal growth two quartiles lower during fetus monitoring, and (3) cerebroplacental ratio (CPR) < the fifth percentile [19].

### 2.4. Hemodynamics and Cardiovascular Parameters

Twenty-four-hour blood pressure (BP) monitoring was performed on all women with a “BPLab” device (Petr Telegin LLC, Nizhny Novgorod, Russia), which meets international accuracy standards for oscillometric BP registration and is recommended for use with pregnant women [20]. BP was measured every 30 min during the day and every 60 min at night. Parameters specific to central BP changes were assessed, i.e., maximal aortal diastolic BP (max DADao); maximal aortal systolic BP (max SADao); minimal aortal diastolic BP (min DADao); minimal aortal systolic BP (min SADao); mean aortal diastolic BP (med DADao); and mean aortal systolic BP (med SADao).

Oscillograms were analyzed using Vasotens software (Petr Telegin LLC, Nizhny Novgorod, Russia). The following parameters of the artery rigidity were analyzed: 1. reflected wave transit time (RWTT): the return time of the wave reflected from the aorta; 2. aortic pulse wave velocity (PWVao): the velocity at which blood pressure pulse propagates in the aorta; 3. augmentation index (AIx): a noninvasive measure of pulse wave reflection; 4. ambulatory arterial stiffness index (AASI: AASI = 1 − (inclination BPdiastolic-BPsystolic); 5. maximal BP increase velocity (dP/dt)_max_: indirectly represents myocardial contractility, total vascular resistance and dynamic load of pulse wave on vascular walls; 6. pulse pressure amplification (PPA): the increase of pulse pressure (PP) amplitude when pressure waves propagate distally in the systemic network, accompanied by morphological alterations of pressure waveforms; 7. ejection duration (ED): an interval of blood flow from the start of pulsation till the closure of the aortic valve; 8. subendocardial viability ratio (SEVR), defined as diastolic to systolic pressure-time integral ratio.

### 2.5. Ultrasonic Dopplerography of Vessels

Dopplerometry of maternal-placental and fetoplacental blood flows was performed by experienced obstetric sonographers using a Voluson E8 ultrasound machines (GE Healthcare Austria GmbH & Co OG Tiefenbach 15 4871 Zipf (Austria)). The uterine artery (UtA), umbilical artery (UA), middle cerebral artery (MCA), and ductus venosus pulsatility indexes (PI) as well as the cerebroplacental ratio were measured. When evaluating the umbilical artery PI, positive or absent diastolic flow was assessed. The ductus venosus was analyzed through cross section and sagittal plane of the fetal abdomen using Doppler color flow mapping. The curve was defined as normal if the A-wave was positive, or abnormal in case of an absent or negative A-wave. 

### 2.6. Detection of Proteoglycan Levels in Blood Serum 

Concentrations of SDC1, HSPG2, and HA were determined in the serum samples collected into vacuum tubes of S-Monovette^®^ Serum, 4.9 mL, cap white, (L × Ø): 90 × 13 mm, with clotting activator. Prior to testing, the samples were centrifuged for 10 min at 2000× *g* and stored at −80 °C. 

Proteoglycan concentrations were determined using enzyme-linked immunosorbent assay (ELISA) kits produced by Antibodies-online.com, Germany (ABIN6730918) for HSPG2; Thermo Fisher Scientific, USA (EHSDC1) for SDC1; and Echelon Biosciences Inc., USA (K-4800) for HA. 

### 2.7. Statistical Analysis

The software used for statistical analysis was “MedCalc version 16.4” (MedCalc, Belgium). For clinical data the distribution normality of the studied traits was assessed using the Shapiro–Wilk criterion. With a normal distribution of the trait, the *t*-test was used (data presented as mean ± standard deviation). Non-normally distributed variables were processed using the Mann–Whitney U test. Data are presented as n (%) for categorical variables, and as a median and an interquartile range for continuous variables. Correlations were investigated by means of scatter plots and the Spearman rank correlation test, and *p* < 0.05 was considered significant.

## 3. Results

### 3.1. Clinical Results

The study of clinical and laboratory characteristics of the patients and their newborns in the studied groups showed that there were no significant differences between the compared groups in age and term of pregnancy at the time of blood sampling. At earlier pregnancy terms (20–34 weeks), significant differences between the groups were found in parity, hemoglobin level, and platelet counts, as well as in the characteristics of the newborns (Table 1). At later pregnancy terms (≥34 weeks), significant differences were found in body mass index (BMI), which was higher in patients with PE, and in birth weight of newborns, which was significantly lower in a complicated pregnancy (Table 1). Patients with early-onset and late-onset PE had a high incidence of chronic arterial hypertension, and a significant number of patients had concomitant FGR.

### 3.2. Assessment of Blood Flow in the Maternal-Placental-Fetal Axis

The main parameters of hemodynamics in the maternal–placental–fetal axis with the assessment of uterine–placental, fetal–placental, and fetal blood flow are shown in Table 2. The following indicators were found to be significantly increased: uterine artery PI in both phenotypes of PE, umbilical artery PI, and cerebro–placental ratio in early-onset PE (Table 2).

### 3.3. Hemodynamic Parameters in Patients with PE and in Normotensive Women of Normal Pregnancy Groups

An analysis of the complex hemodynamic profile showed that daily blood pressure increase was significantly high in both clinical phenotypes of PE. At the same time, the most informative and reliable indicators of arterial stiffness, such as PWVao and RWTT did not change in early-onset PE and late-onset PE (Table 3 and Table 4). However, hemodynamic patterns in early-onset PE and late-onset PE were different. In early-onset PE, there were significant changes in alternative indicators which depend on arterial stiffness: (AIx) or pulse wave increase, which indirectly reflects myocardial contractility—general stiffness of the magistral arteries and dynamic arterial elasticity (dP/dt)_max_. Both indicators had increased, and the latter had a higher significance (Table 3). In late-onset PE, the left ventricular ED and the SERV were higher than in healthy pregnancy. In late-onset PE, the maximum rising speed of left ventricular pressure (dP/dt)_max_ was significantly increased; the AIx and AASI also tended to be increased, with borderline significance compared to normal indicators (Table 4).

### 3.4. Concentrations of Soluble Components of eGC in Maternal Blood

**Figure 1:**
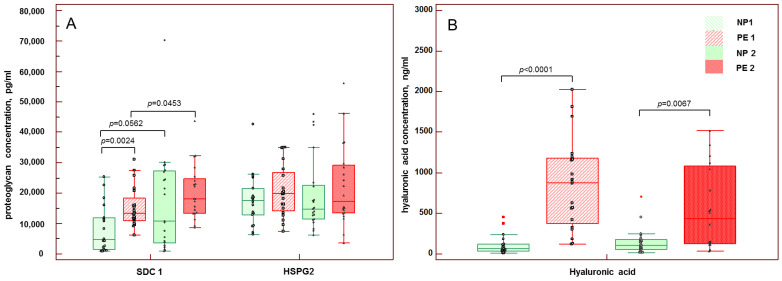
Serum concentrations of syndecan-1 (SDC1), heparin sulfate proteoglycan-2 (HSPG2) (**A**) and hyaluronic acid (**B**) in patients with early-onset PE, late-onset PE and in normotensive women.

It was found that at 20–34 weeks of pregnancy, when the signs of early-onset PE manifested, SDC1 concentration in the peripheral blood was three times higher than in patients with healthy pregnancy: 13,241.6 (10,918.40–18,420.8) pg/mL versus 4301.4 (1580.6–11,637.4) pg/mL (*p* = 0.0024) (Figure 1A). Patients with early-onset PE also had 13.5 times higher HA blood concentration than patients with healthy pregnancy: 879.1 (370.9–1179.4) ng/mL versus 65.0 (37.0–124.8) ng/mL, respectively, (*p* < 0.0001) (Figure 1B). The HSPG2 blood concentration of patients of both groups at 20–34 weeks of pregnancy was comparable: 17,059.0 (13,041.3–20,869.6) pg/mL in healthy pregnancy and 19,796.0 (14,188.3–26,766.0) pg/mL in early-onset PE (*p* = 0.2061) (Figure 1A).

At terms of pregnancy when the symptoms of late-onset PE manifested (≥34 weeks), significant differences were found only in HA concentration; in PE it was more than four times higher than in healthy pregnancy: 437.5 (122.9–1080.9) ng/mL versus 105.0 (51.8–174.3) ng/mL, respectively (*p* = 0.0067). The concentrations of SDC1 and HSPG2 in blood were increased in PE compared to healthy pregnancy: 18,070.4 (13,337.2–24,750.4) pg/mL and 17,275.5 (13,488.8–29,120.03) pg/mL compared to 10,701.6 (3511.2–27,146.8) pg/mL (*p* = 0.1835) and 14,814.0 (11,374.1–22,522.0) pg/mL (*p* = 0.2849), respectively.

The blood concentrations of HSPG2 and HA in healthy pregnancy did not change significantly from earlier (20–34 weeks) to later (≥34 weeks) terms (*p* = 0.7554 and *p* = 0.3229, respectively). However, the SDC1 concentration in healthy pregnancy of later terms was higher and reached the borderline of significance (*p* = 0.0562) (Figure 1A,B).

In early-onset PE, the blood concentrations of HSPG2 and HA tended to be higher than in late-onset PE but did not reach the level of significance (*p* = 0.9569 and *p* = 0.0834, respectively). In contrast, SDC1 concentration was significantly higher in blood of patients with late-onset PE than in patients with early-onset PE (*p* = 0.0453) (Figure 1A,B). 

### 3.5. Correlation of Maternal Hemodynamic Parameters with the Concentration of Soluble Components of eGC in Blood 

**Figure 2:**
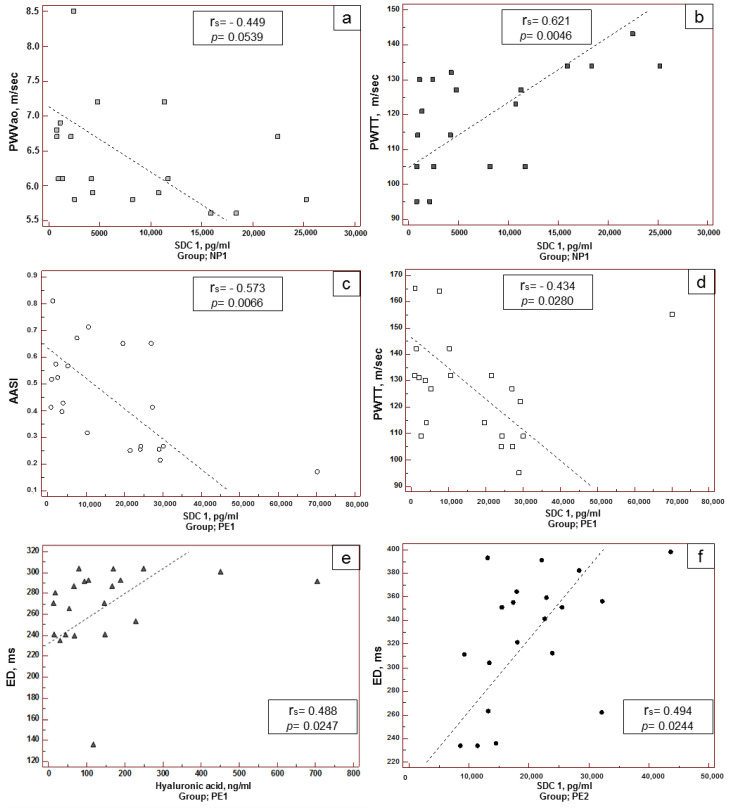
Correlation between proteoglycan/glycosaminoglycan concentrations and hemodynamic parameters in women: with healthy pregnancy of 20–34 weeks (**a**,**b**); with early-onset PE (**c**–**e**); with late-onset PE (**f**). Statistical analysis with Spearman correlation.

There was a direct correlation between blood concentration of SDC1 and RWTT, and a reciprocal correlation with PWVao in patients with healthy pregnancy of 20–34 weeks (Figure 2a,b). Three correlations were found in patients with early-onset PE. In particular, a reciprocal correlation of the concentration of SDC1 with AASI was detected (Figure 2c). Similar to healthy pregnancy, in early-onset PE there was a correlation between SDC1 and RWTT concentrations, but it was reciprocal (Figure 2d). In patients of this group a direct correlation was found between HA and ED (Figure 2e). ED had a direct correlation with SDC1 in the peripheral blood of patients with late-onset PE (Figure 2f). No correlations were found in patients with healthy pregnancies of 20–34 weeks.

Different patterns of correlation between soluble components of eGC in blood and DBPM parameters deserve special attention. In patients with healthy pregnancies at terms of possible development of early-onset PE, strong direct correlations and moderately strong correlation between the concentration of SDC1 and most of the SAD and DAD values were found (Table 5). A moderately strong reciprocal correlation was found between HSPG2 and minimal DAD values. In healthy pregnancies, moderately strong direct correlation was found between the HA concentration and DAD values, and a reciprocal correlation between the HSPG2 concentration and maximum DAD values.

In early-onset PE, a moderately strong reciprocal correlation was found between HSPG2 concentration and DAD values. In late-onset PE, moderately strong reciprocal correlations were noted between the SDC1 concentration, DAD, and SAD values. 

### 3.6. Correlation between Maternal–Placental–Fetal Hemodynamic Parameters and Blood Concentration of Soluble Components of eGC 

**Figure 3:**
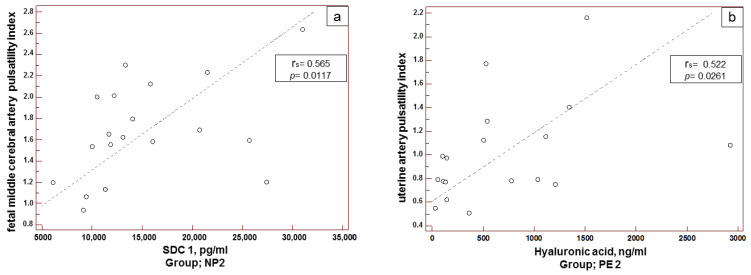
Correlation between proteoglycan/glycosaminoglycan concentrations and dopplerometric parameters in healthy pregnancy of ≥34 weeks (**a**); in late-onset PE (**b**). Statistical analysis with Spearman correlation.

The analysis of correlations between the Doppler measurements in uterine, umbilical, and fetal cerebral arteries and proteoglycan concentration in blood revealed a moderately strong direct correlation between the concentration of SDC1 in blood and MCA-PI in healthy pregnancies of ≥34 weeks (Figure 3a), as well as between the concentration of HA in blood and UtA-PI in late-onset PE (Figure 3b). No significant correlations between the studied parameters were found in patients with healthy pregnancies of 20–34 weeks and early-onset PE.

## 4. Discussion

In this study we investigated two phenotypes of PE related to the disease onset, hemodynamic profiles of women with early-onset PE and late-onset PE, and their correlation with eGC destruction markers (SDC1, HA, HSPG2), considering: (a) the regulatory role of the protective layer of eGC in the processes of mechanotransduction, blood pressure control, vascular permeability, and normal blood cell–vessel wall adhesive interaction [21]; (b) eGC damage with the release of its structural components into the blood, failure of blood pressure control and other homeostatic functions in PE [6,7]; (c) changes in mechanical properties of the arterial wall caused by eGC damage, loss of its structural components and changes in arterial elasticity and stiffness [22]. This study tested the hypothesis of various vascular patterns including hemodynamic changes and different degrees of eGC damage in early-onset PE and late-onset PE. We believe that these processes and conditions are interconnected, can be considered as vascular patterns related to functional disorders and molecular vascular damage, and can be assessed by the hemodynamic parameters and levels of proteoglycans and glycosaminoglycans in the blood.

It is known that maternal hemodynamics undergo significant changes during healthy pregnancy, representing the adaptation of the maternal cardiovascular system to increased blood volume [23]. Altered hemodynamic profiles, compared with healthy pregnancy, are observed in early-onset and late-onset PE [24,25,26,27,28,29]. In the background, there are various pathogenetic mechanisms in early- and late-onset PE, which are caused by placentation pathology and disorders of the fetoplacental blood flow (early-onset PE usually combined with FGR), and maternal somatic pathology in the latent or active phase (late-onset PE). Hemodynamic profiles in early-onset PE have been found to be associated with high total peripheral vascular resistance, low cardiac output, and reduced intravascular volume, while significant cardiovascular disorders usually develop later after delivery. In late-onset PE, high cardiac output occurs in the background of normal or low vascular resistance and hypervolemia [27,28,29]. Previously, it was shown that significant difference in hemodynamic profiles can be explained by association of PE and FGR [30]. Therefore, it was assumed that the cause of hemodynamic differences between early-onset and late-onset PE is probably associated with the development of placental insufficiency. Thus, changes in the hemodynamics of the fetoplacental system can significantly affect overall hemodynamic profile of pregnant women.

Analysis of the clinical data (Table 1) showed that the incidence of FGR was higher in early-onset PE (30%), which confirms the previously published data [30,31], indicating that early-onset PE together with FGR has more severe course and perinatal outcomes. Nevertheless, a significant number of our patients with late-onset PE (20%) also had FGR. The hemodynamic profile of the fetoplacental system showed changes in UtA-PI, UmA-PT, and CPR, which were more pronounced in early-onset PE. These data confirm changes in the patterns of fetal-placental blood flow in early-onset PE, associated with the higher incidence of FGR. However, a correlation between the parameters of uterine and umbilical cord blood flow and eGC components was found only in pregnancies over 34 weeks in both those healthy and complicated by PE. Previously, it was found that fetal glycocalyx of the syncytiotrophoblast and endothelium have significant differences in composition compared to maternal eGC. In particular, the syncytiotrophoblast glycocalyx does not contain HA and heparan sulfate, but has a high concentration of SDC1, which can detach to the blood of pregnant women [32]. However, a decrease in SDC1 expression on the syncytiotrophoblast was found in PE and FGR compared to healthy placenta [33]. Thus, the high level of SDC1 in blood of pregnant women with PE, which was found in our study, does not seem to be associated with shedding from the placenta.

SDC1 in blood can also originate from maternal eGC, since a controlled inflammatory response that develops even in healthy pregnancy stimulates increased shedding of eGC components, which is most pronounced at later terms of pregnancy. In particular, significant amounts of SDC1 are released into the blood during healthy pregnancy and reach maximum by the end of the 3rd trimester [34]; this finding was confirmed in our study. The data on SDC1 concentration in the blood in PE are contradictory: the researchers mostly indicate low concentrations [33], but there are also data on values comparable to those in healthy pregnancy [15,35]. A review of the literature on this topic, undertaken in the framework of our previous study [36], revealed a limited number of studies where SDC1 was determined in patients with PE. In this study, increased SDC1 levels in early-onset PE and significantly higher SDC1 levels in late-onset PE (compared to early-onset PE) were noted. This can be explained by the specific features and small size of this cohort. Another explanation could be the masking of the free form of SDC1 in the blood, probably by antibodies. Our previous studies showed increased levels of antibodies to endothelial antigens [37] and, in particular, to HA and its disaccharide fragments in patients with PE [38]. We did not find any information about antibodies to SDC1 in the cited papers, and have not found any information on them in PE or other pathologies. However, the possibility of such masking cannot be excluded, since hyperactivation of the immune system in PE promotes adaptive immune response to self-antigens, e.g., the most striking example of which are antiphospholipid antibodies [39]. 

Direct correlations between MCA-PI and SDC1 in healthy pregnancy at later terms (≥34 weeks) apparently result from potentially regulatory impact of SDC1 on hemodynamics of the fetoplacental system, since increased blood flow in MCA-PI is a compensatory and adaptive response to hypoxia [40]. A positive correlation between MCA-PI and SDC1 may be caused by the paracrine and autocrine effects of the soluble form of SDC1, or by its competitive activity in some processes, such as regulation of angiogenesis, coagulation, and inflammatory response, [33,41,42] and can facilitate adaptation. Direct correlation between UtA-PI and HA in late-onset PE seems to be associated with adverse outcomes, since an increase in UtA-PI indicates the development of placental insufficiency, and an increase in HA concentration in the blood is evidence of eGC damage, because HA is the basic structural component of eGC, which ensures its stability [8,43]. In our study, increased HA concentrations were observed in patients with early-onset PE and late-onset PE compared to patients with healthy pregnancy at equal terms. This finding is consistent with the data of numerous studies, which are summarized in our earlier publication [36,44], and refer to a similar effect. Therefore, destabilization and damage of eGC structure associated with increased UtA-PI in late-onset PE are a part of an altered vascular pattern in pathological pregnancy.

The key factor, as noted above, that alters the hemodynamics of the fetoplacental system and affects maternal arterial stiffness, is FGR. The studies by Everett, et al. 2012 showed a direct correlation between UtA-PI and arterial stiffness indicators: PWVao, and especially AIx [45]. According to our data, the average direct correlation between UtA-PI and RWTT is detected only in patients with early-onset PE, which confirms a significant contribution of placental insufficiency to changes in the functioning of maternal arteries.

The most commonly used indicator of arterial stiffness is AIx, which changed significantly in patients with PE in our study. AIx represents a composite index of arterial function that is influenced by endothelial dysfunction, arterial compliance, peripheral arterial resistance, and left ventricular ejection [46,47]. In non-pregnant populations, a higher AIx was associated with a higher rate of major adverse cardiovascular events such as myocardial infarction and stroke [47,48]. In pregnancy complicated by hypertensive disorders, especially by PE, AIx was significantly higher than in normal pregnancy, and started to increase from early terms of pregnancy [49,50]; it was also associated with the development of severe PE with concomitant FGR and can serve as an indication for preterm delivery [51]. 

Intracardiac hemodynamic parameters and functional characteristics of myocardium are rarely used in the assessment of vascular status in pregnancy. However, these indicators may be crucial in the assessment of the patient’s condition and prognosis for pregnancy prolongation, particularly in case of concomitant heart disease. The ED is an important indicator of ventricular function as well as ventriculo–arterial coupling, especially for heart failure with reduced ejection fraction [52]. A study by G. Scandale et al. demonstrated that higher myocardial oxygen demand due to left ventricle mass increase is caused by an increase in cardiac workload, wall stress, and left ventricle afterload, which result from the augmentation of the systolic aortic pressure [53]. As a result, the increase of (dP/dt)_max_, which reflects the rate of pressure change in the left ventricle during isovolumic contraction and characterizes left ventricular contractility, indicates an increase in myocardial oxygen consumption and cardiac workload, as well as compensatory changes of the myocardium and its functional characteristics, which are more pronounced in late-onset PE, associated with changes in ED, SEVR, and a trend toward AIx increase.

Our findings show that having similar parameters of central hemodynamics, patients with early-onset PE and late-onset PE have different arterial stiffness and functional indicators of myocardium. In early-onset PE, the signs of arterial stiffness are more pronounced, and are associated with changes in myocardial contractility. Changes in elastic properties of the aorta and loss of its damping characteristics increase the cardiac workload, as observed in these patients. In late-onset PE, stiffness parameters tend to increase, and the impact on myocardial contractility is less pronounced, but signs of myocardial dysfunction are present, as confirmed by prolongation of left ventricular ED. However, patients with late-onset PE had higher subendocardial blood flow velocity and increased left ventricular contractility. Most likely, a shift in these parameters is associated with adaptive changes in coronary arteries and myocardium. 

The features of vascular status described above, including parameters of central hemodynamics, arterial stiffness and myocardial functioning in early-onset PE and late-onset PE, should be considered along with the markers of eGC dysfunction. The analysis of correlations between vascular status and the concentration of eGC components in the blood revealed a reciprocal correlation between SDC1 and PWVao in healthy pregnancy after 34 weeks. Thus, high SDC1 concentration is associated with a decrease in PWVao, and this can be considered a favorable factor contributing to the preservation of arterial wall elasticity. However, the study also showed that in these patients there was a direct correlation between SDC1 and PWTT (which can be interpreted as an unfavorable factor and, moreover, as a contradiction to the reciprocal correlation between SDC1 and PWVao described above), as well as direct correlations with most parameters of central hemodynamics (Table 5). Nevertheless, we do not see any contradiction in these data, because in early-onset PE there was a reciprocal correlation between SDC1 and PWTT (in contrast to that observed normally), and between SDC1 and AASI. It seems that a certain concentration of SDC1 is highly important in pregnancy at earlier terms for maternal vascular homeostasis. SDC1 is also an important factor in late-onset PE: a direct correlation was found between its concentration and prolongation of the left ventricular ED, and a moderately strong reciprocal correlation between the parameters of central hemodynamics was also found (Table 5). SDC1 is a multifunctional molecule that positively and negatively regulates angiogenesis, coagulation pathways, inflammation, lipid metabolism, and the mechanosensing of endothelial cells [33,54,55,56]. Taking into account the studies that suggest a reduced concentration of soluble form of SDC1 in blood can be a risk factor for PE [33], and that its increased concentration can predict the occurrence of multiple organ failure in various pathological conditions [13], the identified correlation between SDC1 concentration and hemodynamic parameters both in healthy and pathological pregnancy shows its dual role and the existence of a concentration–effect relationship.

In early-onset PE, a highly significant reciprocal correlation was found between maximum and average daily DAD values and level of HSPG2 in the blood. In healthy pregnancy, a reciprocal correlation between HSPG2 and minimum and maximum daily DAD values was found in pregnancy at 20–34 weeks and after 34 weeks, respectively (Table 5). Although there were no significant differences in HSPG2 concentrations related to the pregnancy term, its concentration tended to be higher in PE. It seems that the maintenance of a certain stable concentration in blood has a regulatory effect on central hemodynamics of pregnant women. This conclusion is supported by the study of Erkayıran et al., 2021, who concluded that HSPG2 has a protective effect during pregnancy and is a physiologically important molecule for the continuity of the physiological functions in pregnancy [57]. Although this study had a different design, and it was found that the HSPG2 level increased in the severe preeclampsia group in proportion to systolic and diastolic blood pressure, liver and kidney function tests [57], the authors did not link these effects with eGC damage.

The analysis of correlations between certain parameters of vascular status and HA blood concentration, which was increased in PE during the studied terms of pregnancy, also deserves attention. In early-onset PE, we found that prolongation of the left ventricular ejection (ED) was associated with an increase in HA concentration in blood. In healthy pregnancies over 34 weeks, direct correlations between HA concentration and maximum and average daily DAD values were found. It should be noted that biological functions of HA depend on its predominant fraction in the blood. In particular, high-molecular weight HA (HMW-HA, molecular weight > 500 kDa) is the main fraction that is present in a healthy state. HMW-HA regulates vascular homeostasis and has a pronounced anti-inflammatory effect. The low-molecular weight HA (LMW-HA, molecular weight ranging from 10 to 500 kDa), on the contrary, serves as an “danger signal” and has an inflammatory effect [44,58]. The Hyaluronic Acid (HA) Sandwich ELISA test used in our study was able to detect HA with molecular weight > 130 kDa; this means that HMW-HA and LMW-HA could not be distinguished. As a result, we can assume, that HMW-HA and LMW-HA are present in healthy pregnancy and in pregnancy with PE, however, this issue requires further study.

Thus, the findings of the study showed a significant eGC damage in patients with PE, manifesting with an increased amount of circulating structural components of eGC in blood. The signs of eGC damage are more pronounced in early-onset PE, which is evidenced by a significant increase in the blood not only of eGC components with a lack of the transmembrane domain (HSPG2 and HA), but also of SDC1, belonging to the group of transmembrane proteoglycans. An increased concentration of SDC1 in blood should be considered as severe eGC damage, which follows shedding of the outer layer of eGC, and may indicate denudation of the apical membrane of an endothelial cell. SDC1 seems to play an important physiological and pathophysiological role in the homeostasis of the maternal cardiovascular system and vessels of the fetoplacental system, since the changes in its concentration are associated with changes in hemodynamic parameters of these systems both in healthy state and in pathology. In late-onset PE, only the main structural glycosaminoglycan, HA, which performs an organizing and stabilizing function for the entire eGC structure, including ensuring eGC regeneration after damage, was changed. Changes in HA concentration are likely to have greater pathophysiological significance, since they were found in all studied terms of pregnancy and were associated with changes in hemodynamics of the fetoplacental system, central hemodynamics, and myocardial functioning, mainly in PE. 

## 5. Conclusions

It seems that a complex of clinical manifestations of PE develops as a result of significant changes in the molecular composition and structure of eGC. Since eGC integrity correlates with hemodynamic parameters of cardiovascular system, its destabilization leads to formation of adverse molecular and functional vascular patterns. The main outcome of our study was the description of these patterns: (1) in early-onset PE, alterations in all structural components of eGC, including proteoglycans along with the significant disorders in central hemodynamics, signs of arterial stiffness and changes in the myocardium, may indicate a severe course of PE and development of a morphological substrate of a cardiovascular disease and long-term complications; (2) the vascular pattern in late-onset PE is characterized by changes in HA, which, together with the alterations in central hemodynamics and parameters of the myocardium functionality, may indicate the progression of cardiovascular disorders that require careful management during pregnancy. 

Thus, our findings demonstrated that PE may cause a change of adaptive hemodynamic responses caused by pregnancy to pathologic reaction associated with eGC dysfunction. The development of eGC dysfunction has pathophysiological significance and affects the arterial elasticity and the left ventricular myocardium with its subsequent hypertrophy and decompensation, leading to the delayed development of cardiovascular diseases after PE. Further clinical studies of these parameters over the pregnancy course with subsequent follow-up are required to identify predictors of PE and long-term complications of the disease.

## Figures and Tables

**Table 1:** Clinical characteristics of patients included in the study.

	Group				*p*-Values	
Variable	NP1	PE1	NP2	PE2	NP1 vs. PE1	NP2 vs. PE2
n	19	20	21	20		
Maternal age, y ^a^	33.28 ± 1.21	33.60 ± 1.32	34.29 ± 1.08	34.75 ± 1.13	0.8589	0.7680
GA at blood sampling, wk ^a^	29.95 ± 0.64	30.28 ± 0.53	36.61 ± 0.52	37.03 ± 0.33	0.6927	0.5002
Nulliparous ^b^	11 (61%)	4 (20%)	7 (33%)	11 (55%)	**0.0129**	0.1775
BMI, kg/m^2 c^	24.8 (23.0–26.34)	25.20 (23.92–30.88)	26.89 (23.18–28.74)	28.12 (26.45–31.63)	0.3648	**0.0217**
Pre-existing hypertension, n (%) ^b^	0	9 (45%)	0	7 (35%) *	**0.0021**	**0.0054**
PE with FGR, n (%) ^b^	0	6 (30%)	0	4 (20%) **	**<0.0001**	**<0.0001**
Haemoglobin, g% ^c^	120.0 (115.0–124.5)	108.5 (104.5–119.5)	119.0 (112.5–123.5)	121.0 (114.5–125.5)	**0.0075**	0.3322
Thrombocytes (10^9^/L) ^c^	227.50 (207.5–300.0)	176.5 (142.5–220.5)	232.0 (182.5–262.0)	210.5 (178.5–259.0)	**0.0177**	0.7149
Birth weight, g ^c^	3040.0 (2867.5–3348.8)	1313.5 (875.0–1682.0)	3265.0 (3060.0–3528.0)	2479.5 (2120.0–3363.0)	**<0.0001**	**0.0124**
Newborns’ Apgar scores (1 min) ^c^	8 (8–8)	7 (6–7)	8 (8–8)	8 (7.5–8.0)	**0.0002**	0.3391
Newborns’ Apgar scores (5 min) ^c^	9 (8.25–9)	8 (7–8)	9 (8.75–9)	9 (8–9)	**0.0003**	0.3082

^a^ Data are presented as mean ± standard deviation, *t*-test; ^b^ Data are presented as absolute numbers and %, Mann–Whitney u test; ^c^ Data are presented as medians (with interquartile range), Mann–Whitney u test; * comparison between early-onset PE and late-onset PE; *p* = 0.5342; ** comparison between early-onset PE and late-onset PE; *p* = 0.0199.

**Table 2:** Mean blood flow velocity in uterine, umbilical, and fetal cerebral arteries.

	Group				*p*-Values	
Variable	NP1	PE1	NP2	PE2	NP1 vs. PE1	NP2 vs. PE2
n	19	20	21	20		
UtA-PI	_0.66_ 0.76 ^0.87^	_1.20_ 1.41 ^1.66^	_0.66_ 0.75 ^0.86^	_0.77_ 0.88 ^1.15^	**<0.0001**	**0.0448**
UmA-PT	_0.77_ 0.86 ^0.92^	_0.97_ 1.03 ^1.54^	_0.72_ 0.88 ^1.00^	_0.74_ 0.84 ^0.94^	**0.0004**	0.8360
CPR	_1.84_ 2.14 ^2.39^	_0.85_ 1.64 ^1.90^	_1.73_ 1.97 ^2.07^	_1.60_ 1.82 ^2.16^	**0.0037**	0.6217
MCA-PI	_1.78_ 1.88 ^2.20^	_1.28_ 1.62 ^2.01^	_1.30_ 1.60 ^1.95^	_1.35_ 1.51 ^1.68^	0.0836	0.8242

Data are presented as medians (with interquartile range, _low quartile_ Me ^upper quartile^), Mann–Whitney u test; UtA-PI—uterine artery mean pulsatility index; UmA-PT—umbilical artery pulsatility index; MCA-PI—Fetal middle cerebral artery pulsatility index; CPR—cerebro–placental ratio.

**Table 3:** Differences in hemodynamic parameters in patients with healthy pregnancy and patients with early-onset PE.

Hemodynamic Parameters *	NP1, n = 19	PE1, n = 20	*p*-Value
AASI	_0.315_ 0.396 ^0.520^	_0.384_ 0.317 ^0.504^	0.8440
AIx, %	**_−70.5_ −62.0 ^−50.8^**	**_−57.0_ −47.0 ^−33.5^**	**0.0243**
(dP/dt)_max_, mm Hg/sec	**_295.6_ 342.0 ^371.5^**	**_456.0_ 560.5 ^675.5^**	**<0.0001**
ED, ms	_257.3_ 292.0 ^311.5^	_247.6_ 293.0 ^304.0^	0.9775
PPA, %	_108.3_ 123.0 ^155.5^	_113.3_ 123.0 ^137.0^	0.9439
PWVao, m/sec	_5.8_ 6.1 ^6.8^	_6.0_ 6.8 ^7.2^	0.1854
RWTT, m/sec	_105.0_ 123.0 ^131.5^	_121.5_ 128.5 ^134.0^	0.1176
SEVR, %	_78.8_ 96.0 ^115.8^	_84.5_ 101.5 ^163.0^	0.3609
Max DADao, mm Hg	**_80.3_ 82.0 ^84.8^**	**_103.0_ 106.0 ^109.0^**	**<0.0001**
Max SADao mm Hg	**_127.3_ 129.0 ^131.0^**	**_153.5_ 162.0 ^172.0^**	**<0.0001**
Min DADao, mm Hg	**_61.3_ 64.0 ^69.5^**	**_74.0_ 76.0 ^79.0^**	**<0.0001**
Min SADao, mm Hg	**_98.8_ 103.0 ^108.8^**	**_111.5_ 116.5 ^120.5^**	**<0.0001**
Med DADao, mm Hg	**_72.3_ 78.0 ^78.8^**	**_93.0_ 94.5 ^100.0^**	**<0.0001**
Med SADao, mm Hg	**_112.0_ 117.0 ^119.8^**	**_138.0_ 143.0 ^147.5^**	**<0.0001**
Circadian rhythm	**_1.0_ 1.0 ^1.0^**	**_1.0_ 2.0 ^2.0^**	**<0.0001**

* Data are presented as median (Me) with interquartile range (_low quartile_ Me ^upper quartile^), Mann–Whitney U test. AASI, arterial stiffness index; AIx, augmentation index; (dP/dt)_max_, maximal blood pressure increase velocity; ED, ejection duration; max DADao, maximal aortal diastolic blood pressure; min DADao, minimal aortal diastolic blood pressure; min SADao, minimal aortal systolic blood pressure; med SADao, mean aortal systolic blood pressure; med DADao, mean aortal diastolic blood pressure; max SADao, maximal aortal systolic blood pressure; PPA, pulse pressure amplification; RWTT, reflected wave transit time; PWVao, aortic pulse wave velocity; SEVR, subendocardial viability ratio.

**Table 4:** Differences in hemodynamic parameters in patients with healthy pregnancy and patients with pregnancy complicated by late-onset PE.

Hemodynamic Parameters *	NP2, n = 21	PE2, n = 20	*p-*Value
AASI	_0.263_ 0.412 ^0.592^	_0.452_ 0.562 ^0.651^	**0.0621**
AIx, %	_−71.0_ −56.0 ^−32.3^	_−52.5_ −38.0 ^−23.0^	**0.0518**
(dP/dt)_max_, mm Hg/sec	**_266.8_ 370.0 ^456.0^**	**_340.0_ 434.0 ^544.5^**	**0.0446**
ED, ms	**_241.0_ 281.0 ^293.0^**	**_283.5_ 346.0 ^361.5^**	**0.0015**
PPA, %	_110.8_ 119.0 ^137.0^	_121.0_ 127.5 ^142.0^	0.1174
PWVao, m/sec	_5.9_ 6.9 ^7.2^	_6.6_ 7.2 ^8.8^	0.1065
RWTT, m/sec	_109.0_ 127.0 ^134.5^	_111.0_ 118.5 ^130.0^	0.2299
SEVR, %	**_72.0_ 102.0 ^133.0^**	**_115.5_ 129.5 ^152.5^**	**0.0224**
Max DADao, mm Hg	**_79.0_ 81.0 ^86.3^**	**_100.5_ 105.5 ^110.0^**	**<0.0001**
Max SADao mm Hg	**_127.0_ 129.0 ^134.0^**	**_151.0_ 161.5 ^170.0^**	**<0.0001**
Min DADao, mm Hg	**_64.8_ 69.0 ^69.0^**	**_77.5_ 79.5 ^83.0^**	**<0.0001**
Min SADao, mm Hg	**_103.0_ 105.0 ^108.3^**	**_112.0_ 119.0 ^123.0^**	**<0.0001**
Med DADao, mm Hg	**_72.8_ 75.0 ^81.0^**	**_90.0_ 93.5 ^102.0^**	**<0.0001**
Med SADao, mm Hg	**_115.5_ 119.0 ^121.0^**	**_133.0_ 140.5 ^147.0^**	**<0.0001**
Circadian rhythm	**_1.0_ 1.0 ^1.0^**	**_1.0_ 2.0 ^2.0^**	**<0.0001**

* Data are presented as median (Me) with interquartile range (_low quartile_ Me ^upper quartile^), Mann-Whitney U test. AASI, arterial stiffness index; AIx, augmentation index; (dP/dt)_max_, maximal blood pressure increase velocity; ED, ejection duration; max DADao, maximal aortal diastolic blood pressure; min DADao, minimal aortal diastolic blood pressure; min SADao, minimal aortal systolic blood pressure; med SADao, mean aortal systolic blood pressure; med DADao, mean aortal diastolic blood pressure; max SADao, maximal aortal systolic blood pressure; PPA, pulse pressure amplification; RWTT, reflected wave transit time; PWVao, aortic pulse wave velocity; SEVR, subendocardial viability ratio.

**Table 5:** Results of correlation analysis showing the relationship between the changes in the concentration of soluble components of eGC in maternal blood and maternal hemodynamic parameters in patients of all studied groups.

Proteoglycans	Hemodynamic Parameters					
	max DAD	max SAD	min DAD	min SAD	med DAD	med SAD
Group NP1						
SDC-1	0.720; *p* = 0.0005	N.S.	0.641; *p* = 0.0031	0.621; *p* = 0.0046	0.706; *p* = 0.0007	0.601; *p* = 0.0065
HSPG2	N.S.	N.S.	−0.467; *p* = 0.0438	N.S.	N.S.	N.S.
HA	N.S.	N.S.	N.S.	N.S.	N.S.	N.S.
Early-onset PE						
SDC-1	N.S.	N.S.	N.S.	N.S.	N.S.	N.S.
HSPG2	−0.607; *p* = 0.0035	N.S.	N.S.	N.S.	−0.552; *p* = 0.0094	N.S.
HA	N.S.	N.S.	N.S.	N.S.	N.S.	N.S.
Group NP2						
SDC-1	N.S.	N.S.	N.S.	N.S.	N.S.	N.S.
HSPG2	−0.454; *p* = 0.0441	N.S.	N.S.	N.S.	N.S.	N.S.
HA	0.446; *p* = 0.0488	N.S.	N.S.	N.S.	0.459; *p* = 0.0416	N.S.
Late-onset PE						
SDC-1	−0.522; *p* = 0.0181	−0.553; *p* = 0.0155	−0.632; *p* = 0.0028	−0.603; *p* = 0.0049	N.S.	−0.562; *p* = 0.0099
HSPG2	N.S.	N.S.	N.S.	N.S.	N.S.	N.S.
HA	N.S.	N.S.	N.S.	N.S.	N.S.	N.S.

N.S. indicates not significant.

## Data Availability

Data are available upon reasonable request from the corresponding authors.

## Abbreviations

AASI	arterial stiffness index
AC	abdominal circumference
AIx	augmentation index
BMI	body mass index
BP	blood pressure
CPR	cerebroplacental ratio
DAD	diastolic blood pressure
DBPM	daily blood pressure monitoring
(dP/dt)_max_	maximal blood pressure increase velocity
ED	ejection duration
eGC	endothelial glycocalyx
FGR	fetal growth restriction
GA	gestational age
HA	hyaluronic acid
HSPG2	heparan sulfate proteoglycan 2
LMW-HA	low-molecular weight hyaluronic acid
max DADao	maximal aortal diastolic blood pressure
max SADao	maximal aortal systolic blood pressure
MCA	middle cerebral artery
med DADao	mean aortal diastolic blood pressure
med SADao	mean aortal systolic blood pressure
min DADao	minimal aortal diastolic blood pressure
min SADao	minimal aortal systolic blood pressure
NP1	healthy pregnancy of 20–34 weeks
NP2	healthy pregnancy > 34 weeks or onwards
PE	preeclampsia
PE1	early-onset PE
PE2	late-onset PE
PI	pulsatility index
PPA	pulse pressure amplification
PP	pulse pressure
RWTT	reflected wave transit time
PWVao	aortic pulse wave velocity
SAD	systolic blood pressure
SDC 1	syndecan-1
SEVR	subendocardial viability ratio
UA	umbilical artery
UtA	uterine arteries
